# Cone-beam CT image quality improvement using Cycle-Deblur consistent adversarial networks (Cycle-Deblur GAN) for chest CT imaging in breast cancer patients

**DOI:** 10.1038/s41598-020-80803-2

**Published:** 2021-01-13

**Authors:** Hui-Ju Tien, Hsin-Chih Yang, Pei-Wei Shueng, Jyh-Cheng Chen

**Affiliations:** 1 Department of Biomedical Imaging and Radiological Sciences, National Yang- Ming University, B306, Experimental building No. 155, Sec. 2, Linong Street, Taipei, 112 Taiwan; 2 Division of Radiation Oncology, Department of Radiology, Far Eastern Memorial Hospital, New Taipei City, Taiwan; 3 Data Science Degree Program, College of Electrical Engineering and Computer Science, National Taiwan University and Academia Sinica, Taipei, Taiwan; 4 Faculty of Medicine, School of Medicine, National Yang-Ming University, Taipei, Taiwan; 5 Department of Medical Imaging and Radiological Technology, Yuanpei University of Medical Technology, Hsinchu, Taiwan; 6 School of Medical Imaging, Xuzhou Medical University, Jiangsu, China

**Keywords:** Computer science, Radiotherapy

## Abstract

Cone-beam computed tomography (CBCT) integrated with a linear accelerator is widely used to increase the accuracy of radiotherapy and plays an important role in image-guided radiotherapy (IGRT). For comparison with fan-beam computed tomography (FBCT), the image quality of CBCT is indistinct due to X-ray scattering, noise, and artefacts. We proposed a deep learning model, “Cycle-Deblur GAN”, combined with CycleGAN and Deblur-GAN models to improve the image quality of chest CBCT images. The 8706 CBCT and FBCT image pairs were used for training, and 1150 image pairs were used for testing in deep learning. The generated CBCT images from the Cycle-Deblur GAN model demonstrated closer CT values to FBCT in the lung, breast, mediastinum, and sternum compared to the CycleGAN and RED-CNN models. The quantitative evaluations of MAE, PSNR, and SSIM for CBCT generated from the Cycle-Deblur GAN model demonstrated better results than the CycleGAN and RED-CNN models. The Cycle-Deblur GAN model improved image quality and CT-value accuracy and preserved structural details for chest CBCT images.

## Introduction

The techniques of radiotherapy have developed rapidly from three-dimensional radiotherapy to volumetric modulated radiotherapy in recent decades^1–3^. The dose distribution conformed to tumours accompanied by rapid dose falloff to critical organs. Image-guided radiotherapy (IGRT) was used to increase treatment accuracy during the radiotherapy course^4–9^. Cone-beam computed tomography (CBCT) integrated into modern linear accelerators is the most widely used volume imaging system in radiotherapy^10,11^. For the body scan of the X-ray Volumetric Imager (XVI system, Elekta company, Stockholm, Sweden), the average dose was in the range of 0.1–3.5 cGy^12,13^. The artefacts of CBCT, including extinction artefacts, beam hardening artefacts, partial volume effects, exponential edge-gradient effects (EEGEs), aliasing artefacts, ring artefacts, and motion artefacts, influenced the image quality. In addition, noise and scatter are well known to produce additional artefacts^14^. However, the CT values in CBCT images may fluctuate because of scattering contamination, depending on the shape, positioning, size, and inner tissue structure^15,16^. The original CT values of CBCT could not be used for dose calculation unless some correction methods were applied^16–18^. For the multiple purposes of image quality improvement and CT value correction, we adopted the deep learning method to resolve these problems. In recent years, some deep learning methods have been designed to improve image quality in medical imaging. Convolutional neural networks (CNNs) and generative adversarial networks (GANs) are two main kinds of deep learning methods used to improve image quality. Hu et al. proposed a residual encoder-decoder convolutional neural network (RED-CNN)^19^, which was designed to remove noise from low-dose CT. The ground truth data, normal-dose CT, came from the National Biomedical Imaging Archive (NBIA), and the input, low-dose CT, was produced by adding noise into the sinograms simulated from the normal-dose images. The output result of the CT images preserved structural details and reduced image noise.

The generative adversarial network (GAN), defined by Goodfellow et al.^20^, contains two networks: generator and discriminator networks. The generator produced generated images from a convolutional neural network and scored by the discriminator compared to the ground truth images. After the training stage, the generator can generate images closer to the ground truth images. The adversarial loss function of the generator and the discriminator plays an important role in GAN.

GANs are known for their image quality improvement, but the vanilla version has numerous problems, such as non-convergence, model collapse and diminished gradient in the training step, because of Jensen–Shannon divergence between the model distribution and data distribution. To solve these problems, Arjovsky et al.^21^ provided the Wasserstein distance in GANs’ loss function.

Because of 1-Lipschitz, WGAN suffers from the weight clipping problem that the weight may constrain on the boundary of the clipping constraint number. Therefore, Gulrajani et al.^22^ provides an alternative method for enforcing the Lipschitz constraint, called WGAN-GP.

The conditional GAN (cGAN)^23^ is similar to an extension of the vanilla GAN. cGAN adds y between the generator and discriminator as additional information, such as word vectors, images or masks, to constrain the generator to generate the desired image. With the additional information y, the generator may be as similar to the traditional supervised network that we set input data as an image, not just noise.

The U-net^24,25^ architecture is developed for fast and precise object segmentation in a 2D image. With the shortcut in the U-net structure, previous layer characteristics can be transferred to the following layers, and backpropagation can avoid weight decay. That is, because of the shortcut, the key in U-net, we can use parameters to create better images. Kida et al.^26^ took U-net as their model to improve image quality for CBCT. They took low-dose CBCT images as input data and planning computed tomography (pCT) as a ground truth for modelling.

Deblur-GAN^27^ made a blurred image into a clear image. It also designed a blur algorithm to create blurred images as input data. Zhu et al.^28^ recently designed CycleGAN as a more amazing image style transfer. It is unprecedented for the CycleGAN structure that two generators generate different domain images that can serve as inputs to another generator, and two generators can compose each other. In our objective, we can imagine that the model can make CBCT with the FBCT style. Kida et al.^29^ used CycleGAN to synthesize improved CBCT as planning CT to improve the image quality of CBCT for pelvic images with soft tissue and bony structures. The purpose of our study was to improve the image quality of CBCT in truncated chest CT images. Our proposed method combines Deblur-GAN and CycleGAN to achieve more precise image transfer in chest CBCT images.

## Methods

### Data pre-processing

Fifteen breast cancer patients were enrolled in this study. Before radiotherapy, each patient underwent planning CT, that is, the FBCT acquired by a Big Bore CT scanner (Discovery CT590 RT, GE company, Boston, USA), for treatment planning. The acquisition parameters of the GE CT scanner were detector rows of 16, helical scan pitch of 0.938:1, slice thickness of 2.5 mm, and FOV of 50 cm. The adaptive statistical iterative reconstruction (ASiR) algorithm of 30% (SS30) was selected to reconstruct FBCT images. The SS30 denotes the selected ASiR mode as slice statistical reconstruction mode with 30% of the 100% ASiR, which was reconstructed with the original image^30^. The reconstructed FBCT images were used for this study. During every treatment fraction, CBCT was performed for image registration. There are 185 CBCT image datasets, in light of 9856 CBCT images acquired by X-ray Volumetric Imager^10,11^ (XVI system, version R5.0, Elekta company, Stockholm, Sweden) using an optimization of Feldkamp backprojection reconstruction algorithm for training and testing. The acquisition parameters of XVI VolumeView were voltage of the X-ray tube of 120 kVp, current of 40 mA, acquisition time of 120 s, frame rate of 5.5 frames per second, and voxel size of 1 mm × 1 mm × 1 mm for chest CBCT images. An M20 protocol was selected for XVI acquisition, “M” was the FOV of 42.5 cm × 42.5 cm at the kV detector panel, and “20” was 27.67 cm in length at the isocenter of the field projections. FBCT, which was performed once during CT simulation, was used as the ground truth for each CBCT set for this study. Among the images from the fifteen patients, those from three patients (1150 images) were kept for testing, and those from the remaining 12 patients (8706 images) were used to train the network. Pre-processing to build an image pair of CBCT and FBCT was as follows. The CBCT images for each patient were three-dimensionally pre-aligned to each of the FBCT images by rigid registration by PMOD software (Version 3.7, PMOD Technologies, Zurich, Switzerland). To avoid any adverse impact from non-anatomical structures on a CBCT to FBCT registration and as a model training procedure, binary masks were created to separate the body region from non-anatomical regions. These masks were created by finding the maximum convex hull with a threshold CT value of − 1000. CycleGAN does not need paired data, and we could use unpaired images, which may provide various characteristics for model training^28^. To accelerate the model training time, we still used paired data for modelling. We also clipped all image sizes from 512 × 512 down to 264 × 336 to minimize the anatomical region to accelerate the calculation time. We normalized the CT values of CBCT and FBCT images from a range of − 950 to 500 into a range of 0–1. Hence, the pixels with CT values of less than − 950 were assigned to 0, and those with CT values of higher than 500 were assigned to 1. The scale range for modelling is 0–1.

### Image modelling

**Figure 1:**
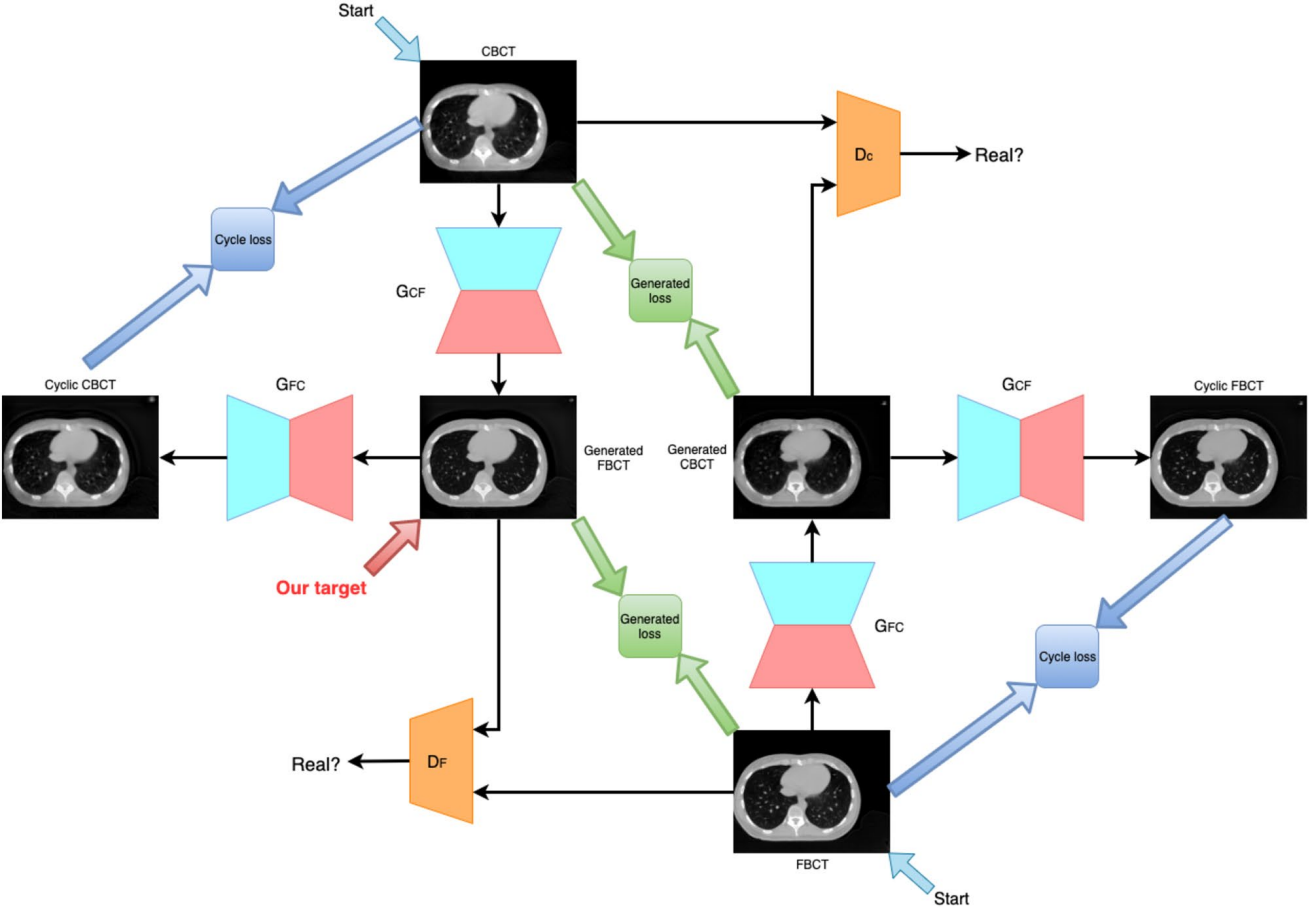
Architecture of Cycle-Deblur GAN.

**Figure 2:**
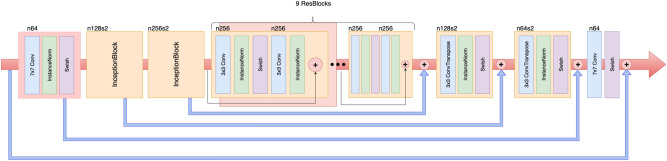
Generator architecture. n denotes the number of convolutional kernels, and s denotes stride. The default stride is 1, i.e., n64s2 denotes the convolution layer of 64 channels with stride 2.

**Figure 3:**
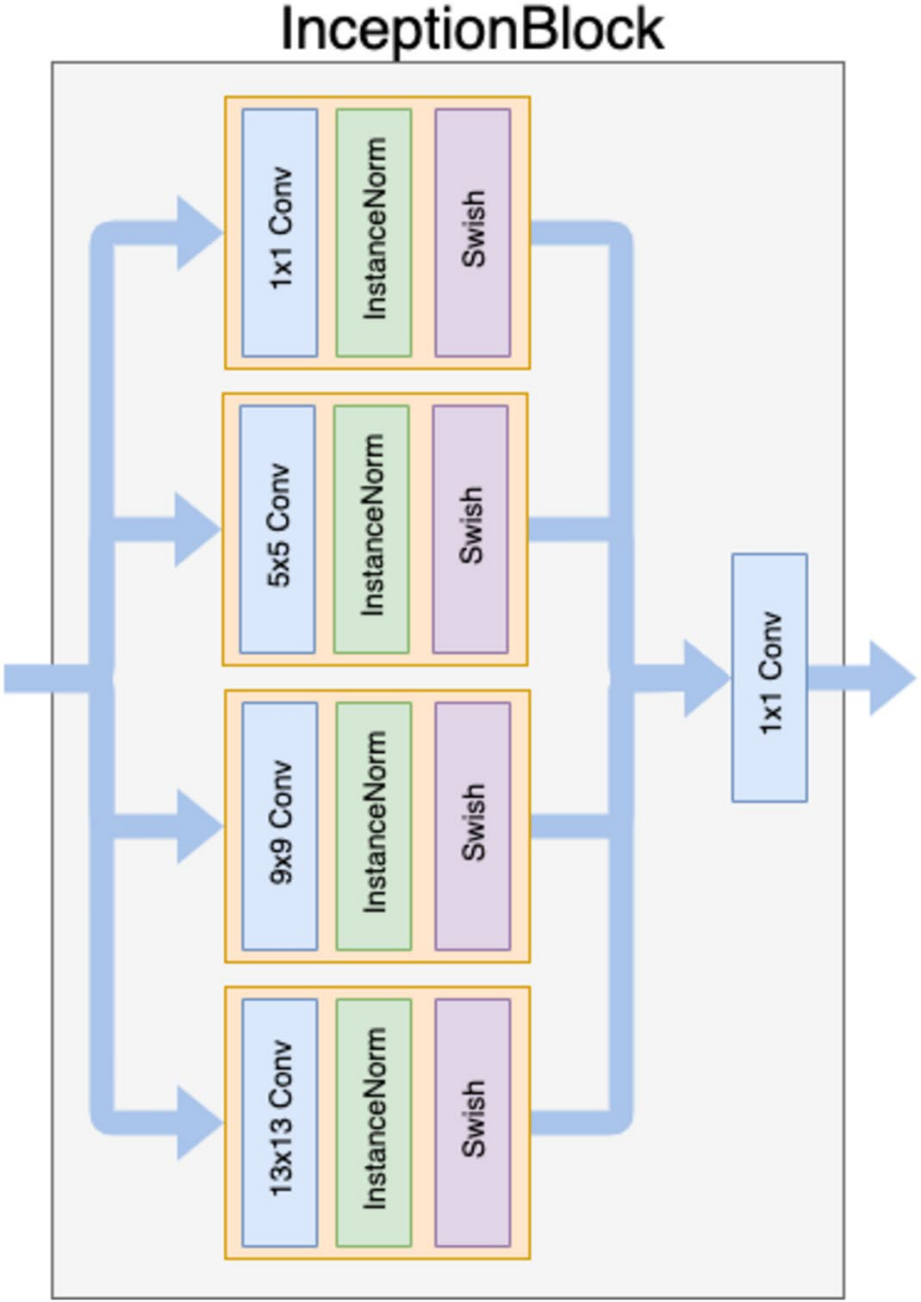
InceptionBlock architecture. It uses different kernel sizes of convolutional layers and deals with detailed and brief features.

An overview of the model architecture is illustrated in Fig. 1. For generator G, we adopted the architecture for our generative networks from Kupyn et al.^27^. They proposed Deblur-GAN, which showed impressive results for generating synthetic clear images from blurred images. The generator architecture is shown in Fig. 2. The inception block^31^ adapted by GoogLeNet can extract different kinds of features by different sizes of convolutions. In our study, the features from the large and small ranges can provide specific results and make the boundary clearer. The generative network contained one convolution block with stride two, two inception blocks with stride two, nine residual blocks and two transposed convolution blocks. Each residual block consisted of a convolution layer, an instance normalization layer, and Swish activation^32,33^. The inception block, a collection of convolution layers of different sizes, such as the 1 × 1 convolution layer, 5 × 5 convolution layer, 9 × 9 convolution layer and 13 × 13 convolution layer, could capture detailed and brief characters without changing the image size. To concatenate different convolution layers in the inception block with different priorities, as shown in Fig. 3, we multiply different weights by 1, 5, 9 and 13.

**Figure 4:**
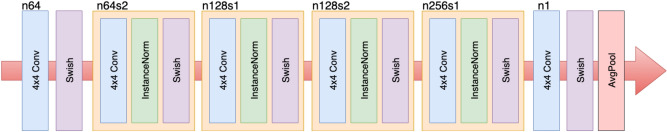
Discriminator architecture.

For discriminator D, shown in Fig. 4, inspired by Ledig et al.^34^, we followed architectural guidelines and dropped the last full connection from the last layer to the convolution layer with the input patch image, which aims to classify whether overlapping image patches were real or generated. Such a patch-level discriminator architecture had fewer parameters than a full-image discriminator and could work on arbitrarily sized images in a fully convolutional method.

### Loss functions

Our goal in this study is to define a deep neural network that finds a suitable mapping function that minimizes the loss functions. Let $x\in X$ and X was CBCT that was an input image. Let $y\in Y$ and Y be FBCT, which was the ground truth image. In general, we can define the mapping function as Eq. (1)

1 $$\widehat{y}={G}_{\theta }(x)$$

where $\widehat{y}$ is the synthesis FBCT image and ${G}_{\theta }$ is the generative model that transforms $x$ to $y$. To obtain decent $\widehat{y}$, the loss function for ${G}_{\theta }$ to generate a synthesis image from the input is shown in Eq. (2)

2 $$\widehat{\theta }=arg\underset{\theta }{\mathrm{min}}\sum_{i}L\left({G}_{\theta }\left({x}_{i}\right), {y}_{i}\right)$$

where $({x}_{i}, {y}_{i})$ are paired CBCT and FBCT images. Inspired by Kida et al.^29^, our model has two generative models ${G}_{CF}:X\to Y$ and ${G}_{FC}:Y\to X$ by input image pairs $({x}_{i}, {y}_{i})$. The two different generative models are trained to synthesize different targets such that ${G}_{CF}$ generates synthesis FBCT by input CBCT; otherwise, ${G}_{FC}$ outputs synthesis CBCT by giving FBCT. Moreover, there are two adversarial discriminators ${D}_{F}$ and ${D}_{C}$, which aim to distinguish whether the output of the generative model is real or synthesis. For example, given FBCT input $y$, ${G}_{FC}$ intends to generate synthesis CBCT $\widehat{x}$, which will be as similar as real CBCT $x$ to foolish discriminator ${D}_{C}$. In contrast, ${D}_{F}$ will judge the reconstructed $\widehat{y}$ from generative model ${G}_{CF}$ by feeding synthesis CBCT $\widehat{x}$ and real $x$. That is, a cyclic method in which the discriminators are not only discriminator synthesized CBCT (or FBCT) but also reconstructed CBCT. The key idea is that generators and discriminators are trained on each other to enhance their accuracy. Therefore, our objective function is a minimax problem, as shown in Eq. (3):

3 $$\underset{G,F}{\mathrm{min}}\underset{{D}_{F}, {D}_{C}}\;{\mathrm{max}}{L}_{gan}\left({G}_{CF}, {D}_{F}\right)+{L}_{gan}\left({G}_{FC},{D}_{C}\right)$$

Additionally, our novel networks include five types of loss functions: adversarial loss (*adv*); cycle-consistency loss (*cycle*); generated loss (*generated*); identity loss (*identity*); and Sobel filter loss (*Sobel*)^35^.

For the discriminators, we adopt WGAN-gp as our objective functions as Eqs. (4) and (5):

4 $$\underset{{G}_{\mathit{CF}}}{\mathrm{min}}\underset{{D}_{F}}\;{\mathrm{max}}{L}_{WGAN-gp}\left({G}_{CF},{D}_{F}\right)={\mathbb{E}}_{x}\left[{D}_{F}({G}_{CF}\left(x\right))\right]-{\mathbb{E}}_{y}\left[{D}_{F}\left(y\right)\right]+\lambda {\mathbb{E}}_{\mathbb{y}}\left[{\left({\Vert \nabla {D}_{F}\left({\mathbb{y}}\right)\Vert }_{2}-1\right)}^{2}\right]$$

5 $$\underset{{G}_{\mathit{FC}}}{\mathrm{min}}\underset{{D}_{C}}\;{\mathrm{max}}{L}_{WGAN-gp}\left({G}_{FC},{D}_{C}\right)={\mathbb{E}}_{y}\left[{D}_{C}\left({G}_{FC}\left(y\right)\right)\right]-{\mathbb{E}}_{x}\left[{D}_{C}\left(x\right)\right]+\lambda {\mathbb{E}}_{\mathbb{x}}\left[{\left({\Vert \nabla {D}_{C}\left({\mathbb{x}}\right)\Vert }_{2}-1\right)}^{2}\right]$$

where ${\mathbb{E}}\left[\bullet \right]$ is the expectation operator, the first two terms are the negative Wasserstein distance, which determines how much better the real term is than the synthesized term, and the last term is the gradient penalty, in which $\lambda$ is a regularization parameter, ${\mathbb{y}}=\varepsilon y+\left(1-\varepsilon \right){G}_{CF}(x)$ and ${\mathbb{x}}=\varepsilon x+\left(1-\varepsilon \right){G}_{FC}(y)$.

Therefore, the overall loss function for the discriminator is shown in Eq. (6):

6 $${L}_{D}={L}_{WGAN-gp}\left({G}_{CF},{D}_{F}\right)+{L}_{WGAN-gp}\left({G}_{FC},{D}_{C}\right)$$

The adversarial loss for generators is as Eq. (7):

7 $${L}_{adv}=-{\mathbb{E}}_{x}\left[{D}_{F}\left({G}_{CF}\left(x\right)\right)\right]-{\mathbb{E}}_{y}\left[{D}_{C}\left({G}_{FC}\left(y\right)\right)\right]$$

Because of adversarial training, discriminators and generators will handle each best. That is, for the loss of discriminators, they encourage real images to score high and synthesized images as low like ${L}_{WGAN-gp}\left({G}_{FC},{D}_{C}\right)$. However, for the generator loss, they intend to let their synthesized images to score much higher, such as $-{\mathbb{E}}_{y}\left[{D}_{C}\left({G}_{FC}\left(y\right)\right)\right]$. Thus, they hold different loss function tasks and alternatively train each other.

The loss function of cycle consistency is shown in Eq. (8):

8 $${L}_{cycle}={\mathbb{E}}_{x}\left[{\Vert x-{G}_{FC}\left({G}_{CF}\left(x\right)\right)\Vert }_{2}\right]+{\mathbb{E}}_{y}\left[{\Vert y-{G}_{CF}\left({G}_{FC}\left(y\right)\right)\Vert }_{2}\right]$$

The loss maps $x\to {G}_{CF}\left(x\right)\to {G}_{FC}\left({G}_{CF}\left(x\right)\right)\approx x$ and $y\to {G}_{FC}\left(y\right)\to {G}_{CF}\left({G}_{FC}\left(y\right)\right)\approx y$, which are referenced to forward cycle consistency loss and backward cycle consistency, respectively.

To make generators able to generate synthesis images, we define the generated loss, which can be expressed as Eq. (9):

9 $${L}_{generated}={\mathbb{E}}_{x,y}\left[{\Vert y-{G}_{CF}\left(x\right)\Vert }_{1}\right]+{\mathbb{E}}_{x,y}\left[{\Vert x-{G}_{FC}\left(y\right)\Vert }_{1}\right]$$

The term can maintain the mapping of $\widehat{y}\approx y$ and $\widehat{x}\approx x$, and it is our final objective. Identity loss is shown in Eq. (10):

10 $${L}_{identity}={\mathbb{E}}_{x}\left[{\Vert {G}_{CF}\left(x\right)-{G}_{CF}\left({G}_{CF}\left(x\right)\right)\Vert }_{2}\right]+{\mathbb{E}}_{y}\left[{\Vert {G}_{FC}\left(y\right)-{G}_{FC}\left({G}_{FC}\left(y\right)\right)\Vert }_{2}\right]$$

The idea for identity loss is that the generative model will transform to the input image style regardless of the input., i.e., given synthesis FBCT to generative model ${G}_{CF}$, the model should still output the image with FBCT style, even if the input is not a real CBCT. A similar method for ${G}_{FC}$ is given synthesis CBCT with the same style output as synthesis CBCT. We use a regularization term to keep the training from overfitting.

Sobel filter loss^35^ is shown in Eq. (11):

11 $${L}_{sobel}={\mathbb{E}}_{x,y}\left[{\delta }_{1}{\Vert y-{G}_{CF}\left(x\right)\Vert }_{1}\right]+{\mathbb{E}}_{x,y}\left[{\delta }_{2}{\Vert y-{G}_{CF}\left(x\right)\Vert }_{1}\right]+{\mathbb{E}}_{x,y}\left[{\delta }_{1}{\Vert x-{G}_{FC}\left(y\right)\Vert }_{1}\right]+{\mathbb{E}}_{x,y}\left[{\delta }_{2}{\Vert x-{G}_{FC}\left(y\right)\Vert }_{1}\right],$$

where ${\delta }_{1}$ and ${\delta }_{2}$ are Sobel gradient operators. The Sobel operator filters the gradient of image colour intensity by ${\delta }_{1}$ and ${\delta }_{2}$ and keeps the edges blurred.

The total objective function for generators can be defined as Eq. (12):

12 $${L}_{G}={\lambda }_{adv}{L}_{adv}+{\lambda }_{cycle}{L}_{cycle}+{\lambda }_{generated}{L}_{generated}+{\lambda }_{identity}{L}_{identity}+{{\lambda }_{sobel}L}_{sobel}$$

The hyper-parameters ${\lambda }_{adv},{\lambda }_{cycle}, {\lambda }_{generated},{\lambda }_{itentity}, {\lambda }_{sobel}$ are changed during the training time, and ${\lambda }_{gan}$ always has a weight of 1. In the first 10 epochs, the loss function is similar to a vanilla GAN, which means that all of the hyper-parameters are 0 except ${L}_{gan}$. In the next 10 epochs, we assigned ${\lambda }_{cycle}=5, {\lambda }_{itentity}=5, { \lambda }_{generated}=1,{\lambda }_{sobel}=0$ as the cycle-consistent period to be the main target. After 20 epochs, we adopt ${\lambda }_{cycle}=10, {\lambda }_{identity}=10,{ \lambda }_{generated}=10,{\lambda }_{sobel}=1{0}^{-4}$ as our main loss function parameters and because if the Sobel gradient loss is too high, the total loss may be misleading; thus, we adopt ${\lambda }_{sobel}=1{0}^{-4}$ as the optimal weight. Our goal is not only for the model to learn style transfer between CBCT and FBCT by cycle consistency but also to make the model fit another style by obtaining direct loss, such as ${\lambda }_{generated}=10$ and ${\lambda }_{sobel}=1{0}^{-4}$. The networks are trained with a learning rate of $1{0}^{-4}$, with the Adam optimizer^36^ and with a batch size of 8. Since GAN has difficulty finding the best minimal loss, we decay the learning rate by a cosine annealing scheduler while keeping the same learning rate in the first 20 epochs.

CT images used for training contain a large range of black backgrounds around the body. The black border will cause the model to be less sensitive to edge pixels. To improve the model stability and prevent overfitting, data augmentation is applied during the training time. Every image pair (CBCT and FBCT), loaded from a dataset, will be synchronously randomly cropped into 128 × 128 sizes. Second, the image pairs are synchronously randomly rotated angles between − 20° and 20° and horizontally and vertically flipped. Then, the image pairs are generated.

We used a personal computer with a single GPU (Nvidia Titan XP) and a CPU (Intel Xeon E5-2620 v4 @ 2.10 GHz) with 64 GB memory, running Ubuntu 18.04 LTS. We implemented our method with Python 3.6.7 and PyTorch 1.0.0. The training time for 200 epochs needs approximately 3 days.

### Quantitative evaluation

**Figure 5:**
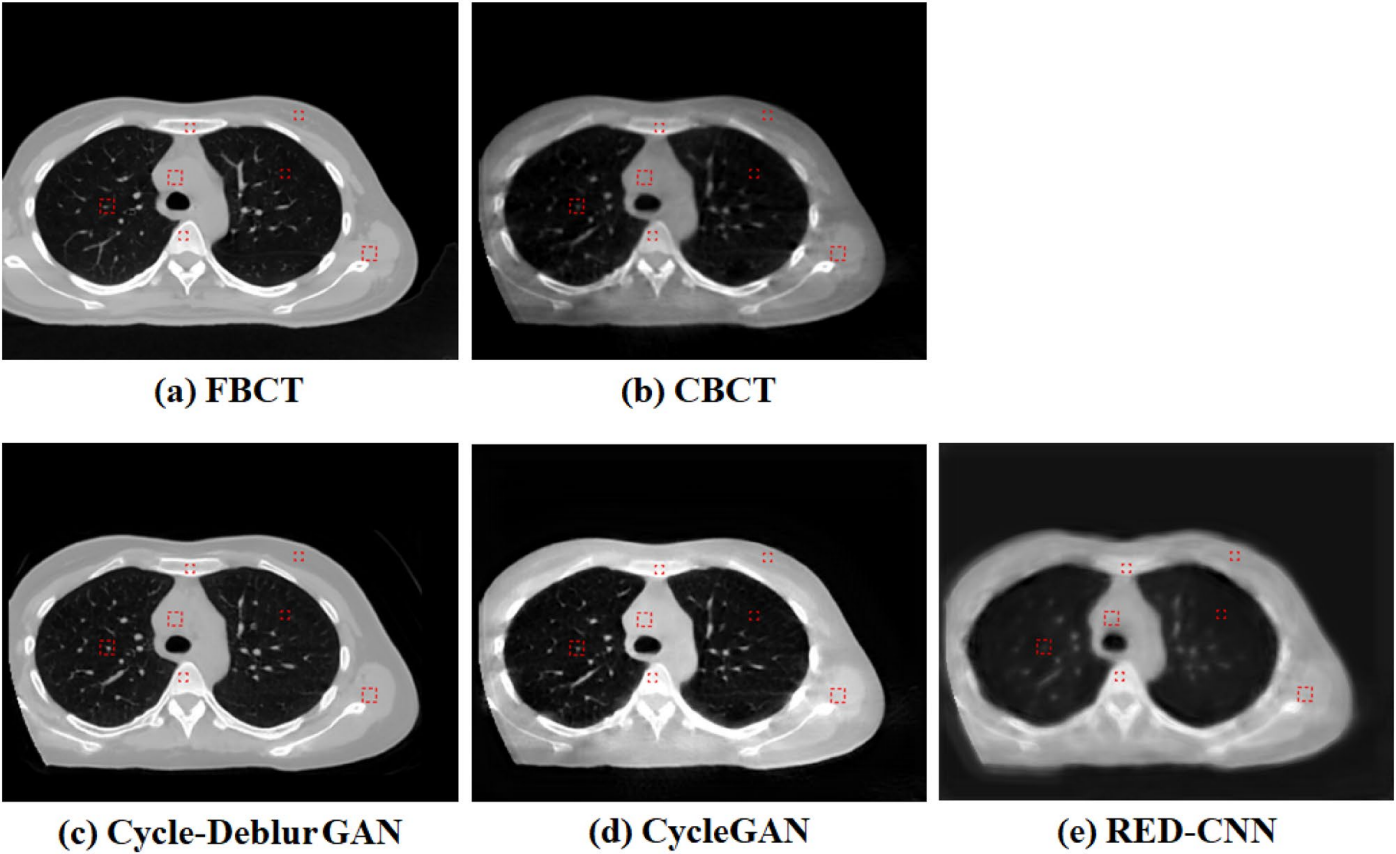
(**a**) FBCT, (**b**) CBCT, and the modeling results of (**c**) Cycle-Deblur GAN, (**d**) CycleGAN, and (**e**) RED-CNN were performed by XVI software and PMOD software. (W = 1450, L = − 225 for all CT images).

The CT value is a linear transformation of the original linear attenuation coefficient measurement into one in which the radiodensity of distilled water at standard pressure and temperature (STP) is defined as the zero CT value, while the radiodensity of air at STP is defined as − 1024 HU. The CT values of the different regions for CBCT, RED-CNN model images, CycleGAN model images, and CycleDeblur GAN images were compared to FBCT. We chose three kinds of soft tissue, breast, muscle, and mediastinum; two kinds of bony structures, sternum and spine; and lung tissue to compare CT values. To evaluate the performance of the proposed method, Cycle–Deblur GAN, we chose existing metrics such as the peak-signal-to-noise-ratio (PSNR), which is measured to capture the reduction in noise, and the structural similarity index measure (SSIM)^37^, which is one of the human visual system-based metrics and to evaluate different attributes such as luminance, contrast, and structure comprehensively. The mean absolute error (MAE) is one of the quantitative evaluations and is also used in our objective (loss) function. The PSNR is calculated from the mean square error (MSE), which is commonly used to measure distortion. There were seven regions of interest (ROIs) shown in Fig. 5, which were used to compare the CT value, MSE, PSNR, and SSIM. We define MSE, PSNR, and SSIM as Eqs. (13)–(21):

13 $$MAE=\frac{1}{N}\sum \left|y-{G}_{CF}\left(x\right)\right|$$

14 $$MSE=\frac{1}{N}\sum {\left(y-{G}_{CF}\left(x\right)\right)}^{2}$$

15 $$PSNR=10\times {\mathrm{log}}_{10} \left(\frac{{MAX}_{I}^{2}}{MSE}\right),\mathrm{ where MAX }=\mathrm{ 65,535 for }16-\mathrm{bit image}$$

16 $$SSIM=\frac{\left(2{\mu }_{x}{\mu }_{y}+{C}_{1}\right)\left(2{\sigma }_{xy}+{C}_{2}\right)}{({\mu }_{x}^{2}+{\mu }_{y}^{2}+{C}_{1})({\sigma }_{x}^{2}+{\sigma }_{y}^{2}+{C}_{2})}$$

17 $${\mu }_{x}=\frac{1}{MN}\sum_{i=0}^{N-1}\sum_{j=0}^{M-1}{x}_{i,j}$$

18 $${\mu }_{y}=\frac{1}{MN}\sum_{i=0}^{N-1}\sum_{j=0}^{M-1}{y}_{i,j}$$

19 $${\sigma }_{x}=\sqrt{\frac{1}{MN-1}\sum_{i=0}^{N-1}\sum_{j=0}^{M-1}{\left({x}_{i,j}-{\mu }_{x}\right)}^{2}}$$

20 $${\sigma }_{y}=\sqrt{\frac{1}{MN-1}\sum_{i=0}^{N-1}\sum_{j=0}^{M-1}{\left({y}_{i,j}-{\mu }_{y}\right)}^{2}}$$

21 $${\sigma }_{xy}=\frac{1}{MN-1}\sum_{i=0}^{N-1}\sum_{j=0}^{M-1}({x}_{i,j}-{\mu }_{x})({y}_{i,j}-{\mu }_{y})$$

### Blind image observer study

The FBCT, CBCT, generated CBCT images from the Cycle-Deblur GAN model, CycleGAN model and RED-CNN model were scored by thirteen medical imaging professionals, including seven radiation oncologists and six medical physicists, using a five-grade scoring method. FBCT was defined as five out of five as the ground truth. The CBCT and generated CBCT images from the Cycle-Deblur GAN model, Cycle-GAN model and RED-CNN model were scored by comparison to FBCT.

### Ethical statement

We confirmed that all methods were carried out in accordance with relevant guidelines and regulations, and informed consent for patients was waived by the Research Ethics Review Committee of Far Eastern Memorial Hospital (FEMH). Images were provided by FEMH and approved by the Research Ethics Review Committee of FEMH (107144-E).

## Results

**Figure 6:**
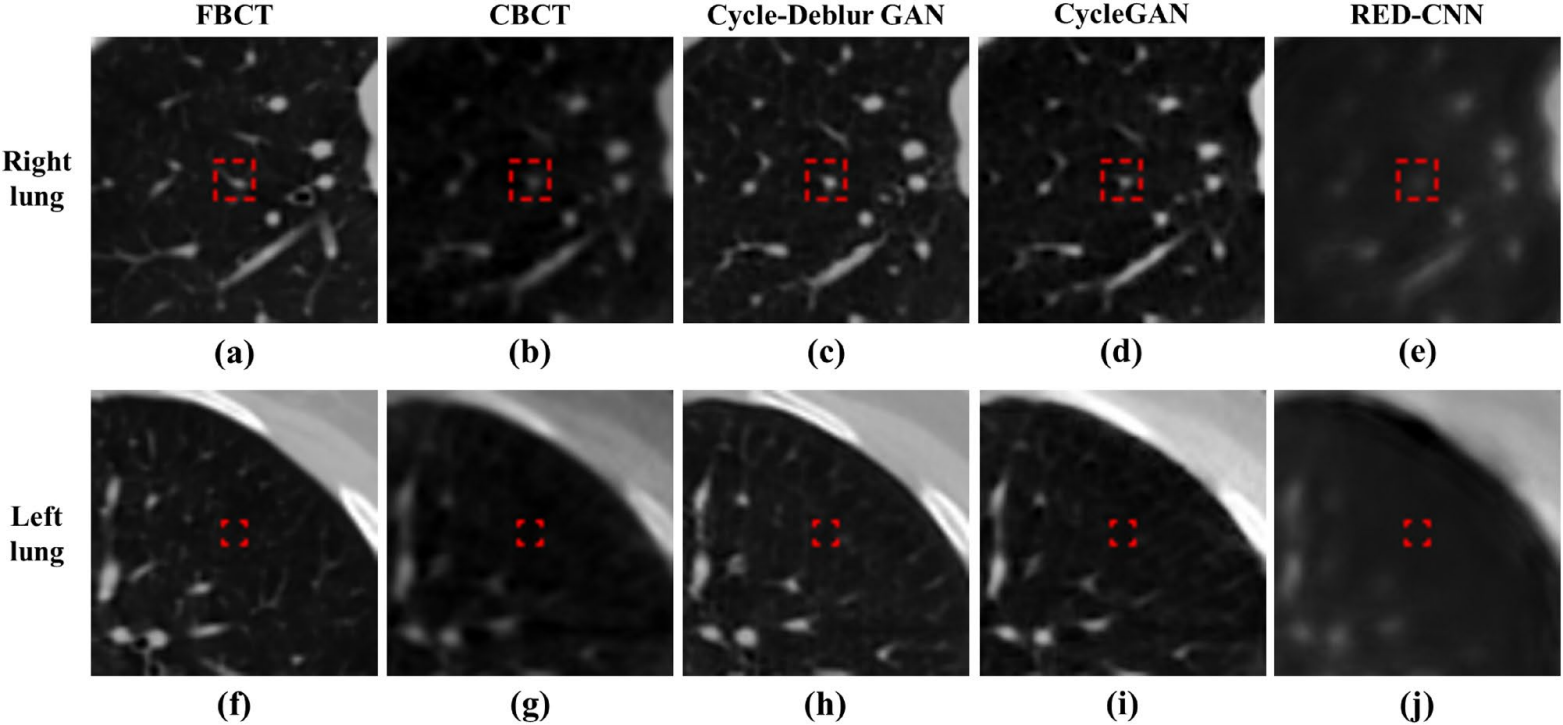
The right and left lung ROIs for quantitative evaluation are shown in (**a**,**f**): FBCT, (**b**,**g**): CBCT, (**c**,**h**): Cycle-Deblur GAN, (**d**,**i**): CycleGAN and (**e**,**j**): RED-CNN were performed by XVI software and PMOD software. (W = 1450, L = − 225 for all CT images).

**Figure 7:**
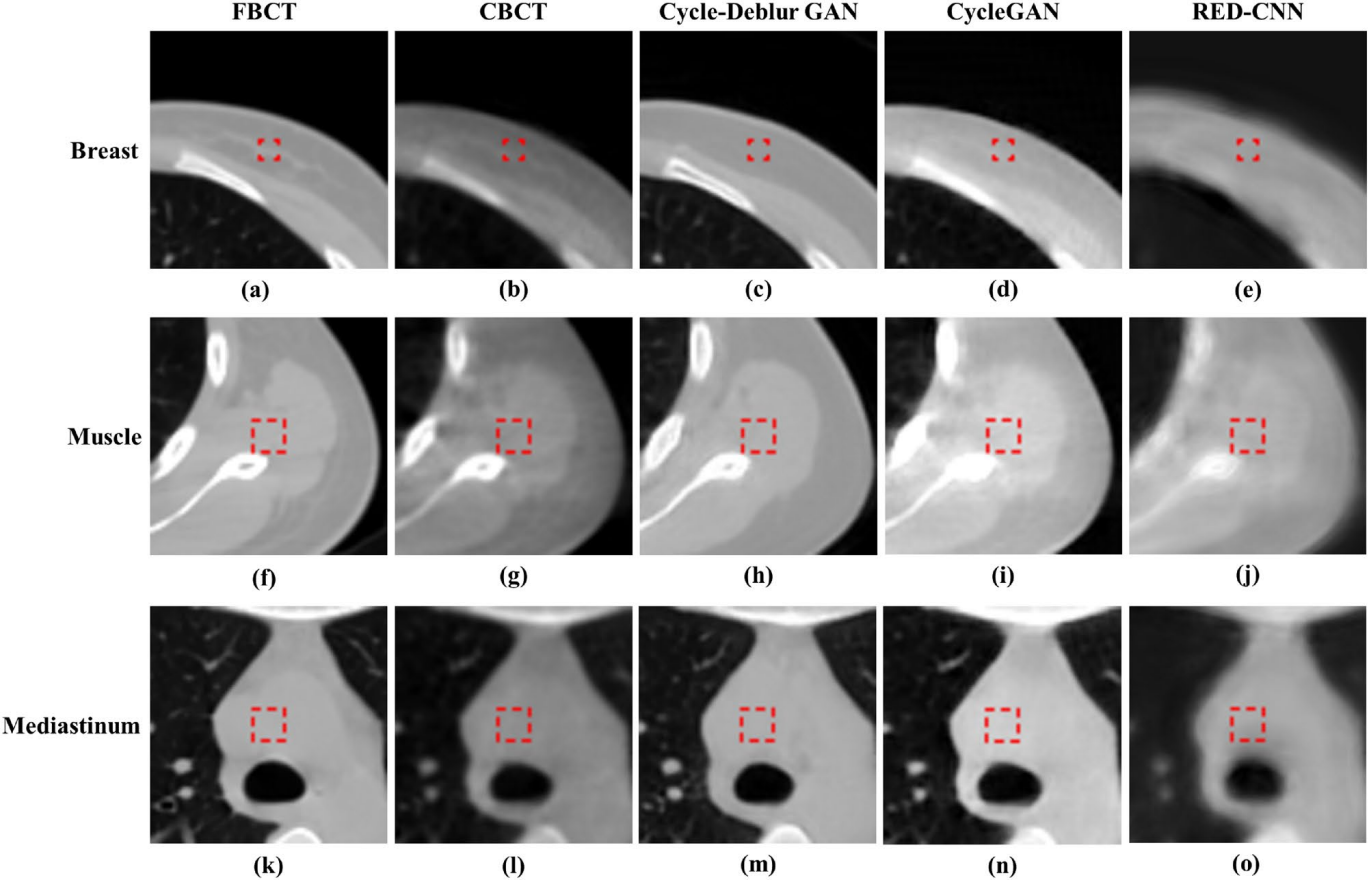
The soft tissue ROIs of the breast, muscle and mediastinum for quantitative evaluation are shown in (**a,f**,**k**): FBCT, (**b**,**g**,**l**): CBCT, (**c**,**h**,**m**): Cycle-Deblur GAN, (**d**,**i**,**n**): CycleGAN and (**e**,**j**,**o**): RED-CNN were performed by XVI software and PMOD software. (W = 1450, L = − 225 for all CT images).

**Figure 8:**
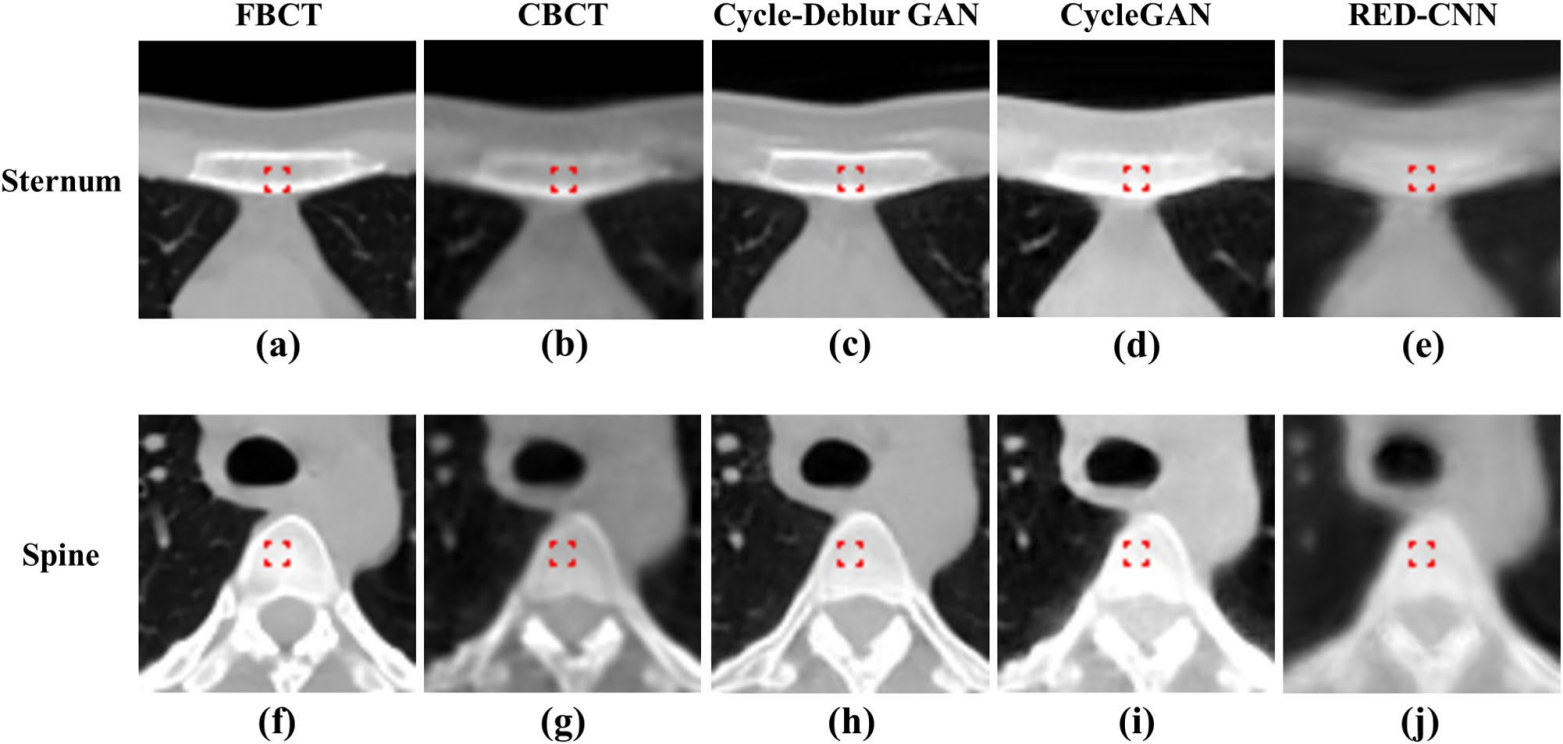
The bony structure ROIs of the sternum and spinal process for quantitative evaluation are shown in (**a**,**f**): FBCT, (**b**,**g**): CBCT, (**c**,**h**): Cycle-Deblur GAN, (**d**,**i**): CycleGAN and (**e**,**j**): RED-CNN were performed by XVI software and PMOD software. (W = 1450, L = − 225 for all CT images).

The generated CBCT images from RED-CNN, CycleGAN, and our proposed Cycle-Deblur GAN with seven ROIs are shown in Fig. 5. The generated CBCT images from Cycle-Deblur GAN performed better visualization than those from CycleGAN and RED-CNN. The CT images of the lung, soft tissue, and bone in seven specific ROIs are shown in Figs. 5, 6, 7 and 8. For the lung ROIs, Cycle-Deblur GAN demonstrated more lung detail preservation than other methods. The ROIs were analysed by the CT value, MAE, PSNR, and SSIM. In Table 1, the CT values of seven ROIs in Cycle-Deblur GAN, CycleGAN, and RED-CNN are shown as the mean values with standard deviations.

**Table 1:** The CT value comparison of different ROIs.

	FBCT (mean ± SD)	CBCT (mean ± SD)	Cycle-Deblur GAN (mean ± SD)	CycleGAN (mean ± SD)	RED-CNN (mean ± SD)
Right lung	− 775 ± 123	− 862 ± 77	− 778 ± 166	− 807 ± 121	− 780 ± 33
Left lung	− 806 ± 34	− 893 ± 33	− 811 ± 28	− 852 ± 39	− 795 ± 16
Breast	− 80 ± 20	− 336 ± 36	− 81 ± 6	26 ± 30	− 42 ± 10
Mediastinum	48 ± 6	− 137 ± 12	40 ± 7	215 ± 12	− 10 ± 45
Muscle	66 ± 9	− 112 ± 23	56 ± 12	238 ± 25	75 ± 25
Sternum	245 ± 151	66 ± 101	230 ± 103	411 ± 52	237 ± 30
Spine	304 ± 19	83 ± 23	308 ± 33	443 ± 27	334 ± 22

The CT values of the ROI in the right lung for FBCT, CBCT, generated CBCT from Cycle-Deblur GAN, CycleGAN, and RED-CNN were − 775 ± 123, − 862 ± 77, − 778 ± 166, − 807 ± 121, and − 780 ± 33, respectively. The CT values of the ROI in breast tissue for FBCT, CBCT, generated CBCT from Cycle-Deblur GAN, CycleGAN, and RED-CNN were − 80 ± 20, − 336 ± 36, − 81 ± 6, 26 ± 30, and − 42 ± 10, respectively. The CT values of the ROI in the sternum for FBCT, CBCT, generated CBCT from RED-CNN, CycleGAN, and Cycle-Deblur GAN were 245 ± 151, 66 ± 101, 230 ± 103, 411 ± 52, and 237 ± 30, respectively. In Table 2, it can be seen that the MAE of our proposed Cycle-Deblur GAN had the smallest value compared to CycleGAN and RED-CNN for different ROIs.

**Table 2:** MAE comparison of different models at different sites.

MAE	Right lung	Left lung	Breast	Mediastinum	Muscle	Sternum	Spine
CBCT	0.06333	0.04346	0.17647	0.12745	0.12325	0.12342	0.15261
Cycle-Deblur GAN	0.03647	0.01242	0.01166	0.00659	0.00894	0.04532	0.01634
CycleGAN	0.03933	0.01427	0.07277	0.11502	0.11804	0.11449	0.09553
RED-CNN	0.04757	0.01841	0.02865	0.04075	0.01510	0.07865	0.02244

In Table 3, the PSNRs for the CBCT, Cycle-Deblur GAN, CycleGAN, and RED-CNN models in the right lung were 21.86, 25.05, 25.38, and 22.88, respectively. The PSNRs of the Cycle-Deblur GAN and CycleGAN models in the lung showed comparable results. The PSNRs for the CBCT, Cycle-Deblur GAN, CycleGAN, and RED-CNN models for breast tissue were 15.01, 36.49, 22.49, and 29.75, respectively. The PSNRs for the CBCT, Cycle-Deblur GAN, CycleGAN, and RED-CNN models for the sternum were 17.74, 25.39, 17.45, and 20.71, respectively. The Cycle-Deblur GAN was shown to have a better PSNR in the breast, mediastinum, muscle, sternum, and spine than the other models.

**Table 3:** PSNR comparison of different models at different sites.

PSNR	Right lung	Left lung	Breast	Mediastinum	Muscle	Sternum	Spine
CBCT	21.86	27.01	15.01	17.87	18.11	17.74	16.28
Cycle-Deblur GAN	25.05	36.07	36.49	40.92	39.18	25.39	32.88
CycleGAN	25.38	35.98	22.49	18.76	18.47	17.45	20.24
RED-CNN	22.88	33.85	29.75	25.64	34.29	20.71	31.25

In Table 4, the SSIM results of CBCT, Cycle-Deblur GAN, CycleGAN, and RED-CNN for the left lung were 0.9922, 0.9993, 0.9992, and 0.9986, respectively. The SSIM results of CBCT, Cycle-Deblur GAN, CycleGAN, and RED-CNN for the sternum were 0.8759, 0.9118, 0.5681, and 0.6849, respectively. The SSIMs of the Cycle-Deblur GAN and CycleGAN models in the lung showed comparable results. The Cycle-Deblur GAN was shown to have better SSIM in the breast, mediastinum, muscle, sternum, and spine than the other models.

**Table 4:** SSIM comparison of different models at different sites.

SSIM	Right lung	Left lung	Breast	Mediastinum	Muscle	Sternum	Spine
CBCT	0.9811	0.9922	0.9134	0.9345	0.9323	0.8759	0.7566
Cycle-Deblur GAN	0.9923	0.9993	0.9972	0.9988	0.9981	0.9118	0.9469
CycleGAN	0.9926	0.9992	0.9726	0.8953	0.8728	0.5681	0.5172
RED-CNN	0.9870	0.9986	0.9924	0.9722	0.9922	0.6849	0.9476

In the blind image observer study, the median years of experience of radiation oncologists and medical physicists were 11 years, with a range from 6 to 33 years, and 8 years, with a range from 6 to 22 years. The results are shown in Table 5. The mean scores of the CBCT and generated CBCT images from the Cycle-Deblur GAN model, CycleGAN model and RED-CNN model were 2.8, 4.5, 3.3, and 1.3, respectively. The CBCT generated from the Cycle-Deblur GAN model scored higher than the other models.

**Table 5:** The results of five-grade scoring method.

	CBCT	Cycle-Deblur GAN	CycleGAN	RED-CNN
RO 1	3	5	4	1
RO 2	2	4	3	1
RO 3	2	4	3	1
RO 4	4	5	4	1
RO 5	2	4	3	1
RO 6	2	4	3	1
RO 7	4	5	3	2
MP 1	4	5	4	3
MP 2	2	4	3	1
MP 3	2	4	3	1
MP 4	2	4	3	1
MP 5	4	5	3	2
MP 6	3	5	4	1
Mean	2.8	4.5	3.3	1.3

*RO* radiation oncologist, *MP* medical physicist.

## Discussion

Our proposed Cycle-Deblur GAN consists of CycleGAN and Deblur-GAN with increasing shortcut numbers and inception blocks to preserve the detailed structure. For the activation layers, since the performance of Swish was better than ReLU in test set accuracy when changing the number of layers^33^, we adopted it as our activation function for Cycle-Deblur GAN. Satoshi Kida et al.^29^ proposed CycleGAN for visual enhancement in pelvic CT images. However, CycleGAN could not perform better to improve image quality in PSNR, SSIM, and MAE for chest CBCT images, as shown in bone and soft tissues. In the RED-CNN^19^ model, 14 input images and ground truth images were created from the same projections for comparison. However, in our study, CBCT and FBCT were acquired from one patient on different days. The image registration was needed before modelling. When using the RED-CNN model to train CBCT and FBCT in our study, the misalignment influenced the results of the RED-CNN model, which showed blurred results. For the Cycle-Deblur GAN model, the CBCT and FBCT images were both treated as the input images to derive a more stable model. Hence, the registration error due to the different acquisition dates of FBCT and CBCT for Cycle-Deblur GAN represented less influence, and the Cycle-Deblur GAN model could generate higher image quality images.

CT values of the original CBCT images may fluctuate for the same material in the different relative positions being scanned in the image volume^15^. In Table 1, the CT values of the generated CBCT from Cycle-Deblur GAN showed better results than those from RED-CNN and CycleGAN in the breast, lung, muscle, mediastinum, and sternum. For bone tissue, including the spine and sternum, the CBCT generated from the RED-CNN model showed a better result in the quantitative analysis of PSNR and SSIM. However, the visual enhancement of the generated CBCT from the RED-CNN model, as shown in Fig. 8, was blurred. The ROI size of the spine, breast, and sternum was smaller than others due to contouring the same structure in one ROI. The PSNR and SSIM of our proposed method demonstrated better results than other methods and showed more detail preservation, especially in lung tissues, as shown in Fig. 6.

Once the Cycle-Deblur GAN was well trained, the generator of the Cycle-Deblur GAN was used for testing. In the testing process, we input the CBCT image passing through the generator model and receive the generated CBCT. The generated CBCT with high image quality benefits the image verification by the oncologist. The average time of the generator to produce an improved CBCT image was approximately 0.17 s and depended on the hardware used.

## Limitations of the study

We proposed the Cycle-Deblur GAN method to model chest CBCT images and obtain better results than the CycleGAN and RED-CNN methods in this study. The input data were all chest CT images, and the modelling generator used to produce the generated CBCT may be limited to the chest region only. Smoothed images of better PSNR with lower noise may be accompanied by lower contrast. Hence, the visualized evaluation of SSIM was also evaluated in our study.

## Conclusions

The CBCT generated by our proposed Cycle-Deblur GAN model demonstrated higher PSNR and SSIM results in soft tissue, lung, and bony structures with improved image quality. The generated CBCT images with accurate CT values can be used for adaptive dose calculation in radiotherapy. The overall artefact of CBCT was well removed by using this model. This model enhanced the structural details in the lung, soft tissue, and bony structure and showed better visualization than the original CBCT. The Cycle-Deblur GAN model improved the image quality of CBCT, preserved structural details and provided accurate CT values for dose calculation. The high image quality and accurate CT values of CBCT would assist the development of radiomics in our future work.

## Data Availability

The datasets used and/or analysed during the current study are available from the corresponding author on reasonable request.
